# A Stereotactic Probabilistic Atlas for the Major Cerebral Arteries

**DOI:** 10.1007/s12021-016-9320-y

**Published:** 2016-11-21

**Authors:** Tora Dunås, Anders Wåhlin, Khalid Ambarki, Laleh Zarrinkoob, Jan Malm, Anders Eklund

**Affiliations:** 1 Department of Radiation Sciences, Umeå University, S-901 87 Umeå, Sweden; 2 Umeå Center for Functional Brain Imaging, Umeå University, S-901 87 Umeå, Sweden; 3 Centre for Biomedical Engineering and Physics, Umeå University, S-901 87 Umeå, Sweden; 4 Department of Pharmacology and Clinical Neuroscience, Umeå University, S-901 87 Umeå, Sweden

**Keywords:** Cerebral arteries, Probabilistic atlas, 4D flow MRI, Automatic labeling, Spatial normalization

## Abstract

Improved whole brain angiographic and velocity-sensitive MRI is pushing the boundaries of noninvasively obtained cerebral vascular flow information. The complexity of the information contained in such datasets calls for automated algorithms and pipelines, thus reducing the need of manual analyses by trained radiologists. The objective of this work was to lay the foundation for such automated pipelining by constructing and evaluating a probabilistic atlas describing the shape and location of the major cerebral arteries. Specifically, we investigated how the implementation of a non-linear normalization into Montreal Neurological Institute (MNI) space improved the alignment of individual arterial branches. In a population-based cohort of 167 subjects, age 64–68 years, we performed 4D flow MRI with whole brain volumetric coverage, yielding both angiographic and anatomical data. For each subject, sixteen cerebral arteries were manually labeled to construct the atlas. Angiographic data were normalized to MNI space using both rigid-body and non-linear transformations obtained from anatomical images. The alignment of arterial branches was significantly improved by the non-linear normalization (*p* < 0.001). Validation of the atlas was based on its applicability in automatic arterial labeling. A leave-one-out validation scheme revealed a labeling accuracy of 96 %. Arterial labeling was also performed in a separate clinical sample (*n* = 10) with an accuracy of 92.5 %. In conclusion, using non-linear spatial normalization we constructed an artery-specific probabilistic atlas, useful for cerebral arterial labeling.

## Introduction

Cerebrovascular imaging is critical in diagnosing several neurological disorders (Kronzon and Tunick 2006; Mueller et al. 2005) as well as in research investigating cerebrovascular physiology and pathophysiology (Amin-Hanjani et al. 2015; Muller and Van Der Graaf 2012; Rivera-Rivera et al. 2015; Zarrinkoob et al. 2015). Several recent technological advancements, embodied in 4D flow MRI, allow for acquisition of data such as velocity, flow rate, turbulence patterns and pulsatility in brain arteries (Frydrychowicz et al. 2011). Therefore, post-processing methods that can transform these datasets into meaningful standardised quantitative descriptions of blood flow in the individual will soon be needed.

Probabilistic tissue maps are commonly used for tissue segmentation in the whole brain (Ashburner and Friston 2005) and in specific areas such as the cerebellum (Diedrichsen et al. 2009; van Baarsen et al. 2016) in both humans and animals (Love et al. 2016). However, such methods have not been explored for labeling and segmentation of cerebral arterial branches.

An atlas describing the spatial distribution of individual arteries could enable a high degree of automation in applications that require labeling of vascular segments. Pioneering work has provided detailed descriptions on individual segments (Nowinski et al. 2009a, b, 2013), as well as probabilistic information on the morphology of the cerebrovascular tree (Forkert et al. 2013; Mut et al. 2014). Recently, we combined these two properties into a probabilistic artery-specific atlas and an automatic, atlas-based artery-identification method (AAIM) (Dunås et al. 2016). The promising results from that proof-of-concept study motivate the construction of an atlas based on a large population-based sample, which would give a more representative and improved spatial coverage of the anatomic variation between individuals.

To validate such an arterial atlas in a meaningful way, the purpose of the atlas must be considered. Therefore, atlases developed for segmentation and labeling are generally evaluated for that specific task (Dunås et al. 2016; Forkert et al. 2013; Passat et al. 2005). Another important evaluation is based on describing the underlying spatial alignment of the structures that are included in the atlas.

The aim of this study was to create a stereotactic and probabilistic cerebral arterial atlas by manually labeling 167 high-resolution 4D flow MRI angiographic scans. This atlas was validated based on its applicability for arterial labeling, and the impact of the normalization process was investigated by comparing non-linear normalization with rigid-body alignment.

## Materials and Methods

In this study, 2360 cerebral arteries were manually labeled in 4D flow MRI from 167 healthy elderly subjects and used to create the atlas. The workflow had five main steps: 1. Data acquisition; 2. Spatial image normalization and preprocessing; 3. Atlas construction; 4. Comparison to rigid-body alignment; and 5. Atlas validation.

### 1. Data Acquisition

#### Subjects from the Population

COBRA (*Cognition, Brain and Aging*) (Nevalainen et al. 2015) is a large, population-based, prospective MR imaging study. In summary, subjects between 64 and 68 years of age were randomly selected from the population registry of Umeå, Sweden. Subjects with medical conditions or medical or surgical interventions that could alter brain function or cognitive performance such as history of brain trauma or stroke, dementia, diabetes, functional impairment or movement disorders (e.g., Parkinson’s disease), epilepsy, intellectual disability, psychological disorders, and ongoing malignancy treatment, were excluded, as well as subjects with contraindications for MRI.

As a part of the COBRA study, we collected 4D flow MRI data from 181 subjects (age 66.2 ± 1.2, M = 100, F = 81). Out of these, thirteen subjects were excluded due to constraints regarding data quality (e.g. motion artifacts) and one due to a vascular malformation. This resulted in a sample of 167 subjects (age 65.8 ± 1.2, M = 97, F = 70) on which the atlas was based.

#### Clinical Sample

For a pilot test on a clinical sample, ten patients with transient ischemic attacks (*n* = 6) or lacunar infarcts (*n* = 4) were included. This diagnosis was based on case history, neurological and brain MRI examination. All patients were also investigated with CT angiography (0.6 mm slices). CT did not reveal any stenosis or occlusion of internal carotid, vertebral or basilar arteries, or in the middle, anterior or posterior cerebral arteries.

#### MRI

The MRI data used in this study were collected on a 3 Tesla scanner (Discovery MR 750; GE Healthcare, Milwaukee, WI, USA) with a 32-channel head coil, using a balanced 5-point 4D flow MRI (Johnson and Markl 2010) covering the intracranial cavity. The scan time for the 4D flow sequence was approximately nine minutes. Imaging parameters were: velocity encoding, 110 cm/s; TR/TE, 6.5/2.7 ms; flip angle, 8°; bandwidth, 166.67 kHz; radial projections, 1600; acquisition resolution, 300 × 300 × 300; imaging volume, 220 × 220 × 220 mm; reconstruction matrix size, 320 × 320 × 320 (zero padded interpolation); and voxel size 0.7 × 0.7 × 0.7 mm^3^. From the 4D flow MRI, a structural magnitude image (M_T1w_) and an angiographic image (AI) were reconstructed (Dunås et al. 2016).

### 2. Image Processing

#### Normalization

Tissue probability maps for white matter, grey matter and cerebrospinal fluid were generated from the structural M_T1w_ using SPM8’s *New Segment*. From these tissue probability maps, a study-specific brain template was generated using SPM8’s DARTEL (Ashburner 2007). In that process, a subject-specific transformation field that described the nonlinear transformation from each subject to the study template was also generated. With these transformation fields, the AI of each subject was transformed to match the group template and then normalized to stereotactic Montreal Neurological Institute (MNI) space by aligning it with the MNI152-template (Evans et al. 2012) using an affine transformation.

#### Vascular Segmentation and Skeleton Construction

The AI was smoothed with a low-pass box filter with a kernel size of three voxels. To give complete vessel coverage without including neighboring static tissue (Wåhlin et al. 2012), the image was binarized by thresholding at 18 % of the maximum intensity value.

**Fig. 1:**
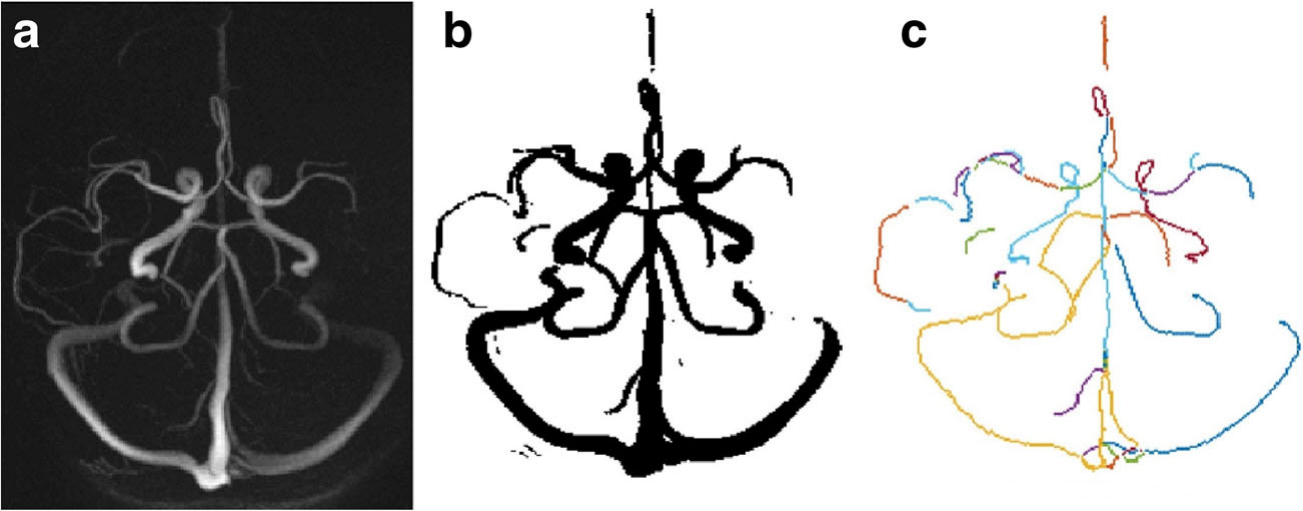
Vascular segmentation and skeleton construction, **a**) raw angiographic image, **b**) segmented vasculature, **c**) vascular skeleton where different branches have different colors

To separate the branches of the vascular tree a vascular skeleton was extracted from the binary image using an automatic method containing three steps: 1. The binary vessel tree was gradually thinned until a one-voxel thick skeleton was obtained (Palàgyi and Kuba 1998); 2. The vascular skeleton was pruned to remove loops and branches shorter than eight voxels; 3. The vascular skeleton was divided into branches separated by junction-points, and each branch was assigned an identification number (Chen and Molloi 2003). Figure 1 illustrates the segmentation and skeleton construction.

### 3. Atlas Construction

#### Included Arteries

The arteries included in the atlas were selected based on their fundamental role in the cerebral arterial circulation. The included arteries were: left and right internal carotid artery (ICA); basilar artery (BA); left and right vertebral artery (VA); left and right posterior cerebral artery (PCA); left and right, proximal and distal middle cerebral artery (MCA); left, right and distal anterior cerebral artery (ACA); and left and right posterior communicating artery (PCoA).

#### Manual Arterial Labeling

The arteries were manually labeled using an in-house tool developed in Matlab (Mathworks, MA, USA). The vessels of the brain were visualized as a rotatable 3D volume. The arterial segments forming each artery were selected, and the corresponding vascular skeleton branches were labeled and saved. The border between two arteries was primarily defined according to junction points in the vascular skeleton. When no such junction points were present (e.g. due to an absent branch) the border was manually determined based on morphology such as changes in diameter or direction (Osborn 1999).

**Fig. 2:**
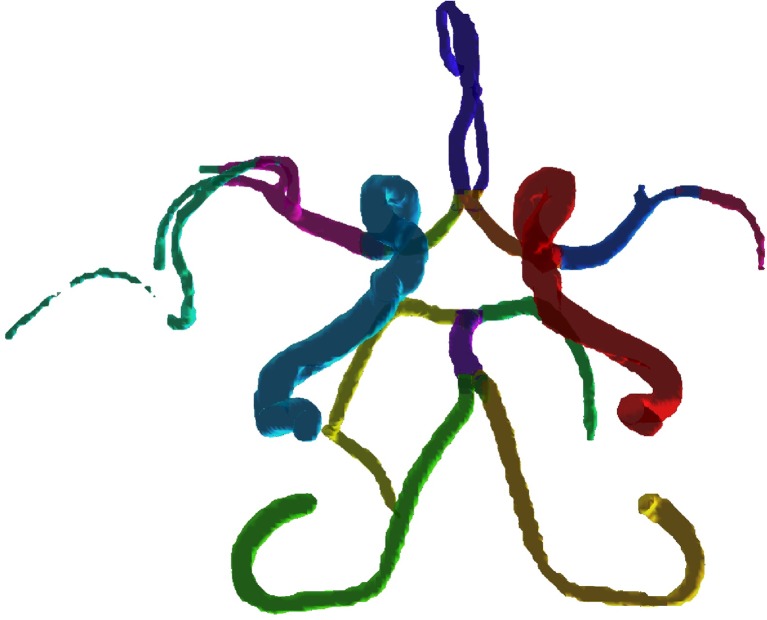
An example of manually labeled arteries for one subject. For ACA_distal_, all main branches distal to the anterior communicating artery were selected (1–3 branches of the pericallosal artery depending on morphology, A2–A3 level). PCA was cropped at P3 level, distal to pons, to get a uniform length (Osborn 1999). MCA was divided into a proximal (MCA) and a distal (MCA_distal_) part. The proximal part consists of the M1 segment, pre- and post-bifurcation. The MCA_distal_ includes the full visible length of MCA, or until it reaches the cortex (M2 and M3 segments). The border between the proximal and distal part was set at the genu where the MCA takes a turn in the posterior direction (Osborn 1999). Only the branches that extend posteriorly (M2) and laterally (M3) were included. For M1, branches forming/preceding the main M2 branches, or having the same direction as those doing so, were included. The direction and continuity of the arteries were decided by visual inspection. Since MCA_distal_ consists of several branches, the individual variation at M3 level was too large for it to be useful to construct a separate probability map. Note that in the vascular segmentation process, gaps sometimes arise in low-flow arteries, here seen in the MCA_distal_ on the left side of the figure

To re-inflate the labeled arteries, the vascular skeleton branches with the corresponding labels were dilated with a kernel of size seven voxels, and the resulting volume was multiplied with the binary vessel tree. The labeled arteries were then visually inspected and approved by a neurologist (LZ). When there was uncertainty regarding which branches that should be included (primarily in MCA), a consensus decision was made by LZ and TD (5 and 3 years of experience in neurovascular anatomy). An example of manually labeled arteries from one subject is presented in Fig. 2.

A probability map was constructed for each artery by adding together the binary volumes of the re-inflated arteries for all subjects and dividing the value of each voxel by the number of included arteries. This resulted in a 3D volume with values between zero and one, corresponding to the proportion of included arteries that overlapped in each voxel. These 16 probability maps together form the atlas, which was denoted Umeå Brain Arteries (UBA167).

### 4. Impact of Normalization

#### Rigid-Body Atlas

To determine the contribution from the DARTEL normalization, the UBA167 was compared with an atlas where the vascular trees were aligned using a rigid-body transform. The obtained volumes were transformed to native space and aligned to the MNI152-template using a rigid-body transform in SPM8. This procedure was required since the manual labeling of each subject was performed after DARTEL normalization. Probability maps for each artery were calculated in the same way as for UBA167.

Since the arteries were extracted after DARTEL normalization, the arterial volumes had to be transformed back to native space before aligning them to the MNI-template in order to create the rigid-body atlas. Due to partial volume effects, the images had to be re-binarized after the rigid-body alignment. This was done using a threshold adapted for volume conservation of each probability map.

#### Atlas Characterization

The normalization procedure was evaluated by comparing the UBA167 to the rigid-body atlas. Four measurements were used to describe the spatial distribution of arteries in terms of alignment between subjects and separation between arteries in the probability maps. For each artery probability map, the measurements were: 1. Concatenated volume calculated from the number of non-zero voxels, i.e. voxels occupied by an artery in any of the 167 subjects; 2. The arterial volume ratio (AVR) obtained by dividing the concatenated volume of the probability map by the average arterial volume across the included subjects; 3. The dominating volume of each probability map calculated as the percentage of voxels where the artery in question had higher probability than any other overlapping artery; 4. The maximum value of each probability map.

A low AVR equals a high spatial agreement between subjects, and hence a high specificity for separating arteries from background. The dominating volume describes how well the probability maps are separated, which could be translated to the specificity for separating different arteries.

### 5. Atlas Validation

#### Leave-One-Out Validation

To evaluate the sensitivity of the UBA167, it had to be applied to a new sample of subjects. This was done using a leave-one-out approach where a target subject was removed from the UBA167 and the modified atlas based on the remaining 166 subjects was used to label the arteries of the target subject, using the previously described automatic identification method (AAIM) (Dunås et al. 2016). This process was repeated for all subjects in the cohort. The labeled images were approved or disapproved according to the following criteria:

- ICA: 2 cm cervical segment
- VA: 1 cm straight segment in conjunction to the foramen magnum
- BA: 1 cm segment anywhere in the artery
- PCA: 1 cm segment at P2–P3, i.e. distal to PCoA
- MCA: 1 cm segment in M1
- MCA_distal_: 1 cm segment in the sylvian fissure
- ACA: 5 mm segment in A1, proximal to the anterior communicating artery
- ACA_distal_: 1 cm segment distal to anterior communicating artery
- PCoA: 5 mm segment anywhere in the artery

Agreement between automatic and manual labeling was described and divided into six categories:

- Correctly identified existing: The artery was present in the manual reference and was localized.
- Correctly identified nonexisting: The artery was not present in the manual reference and was marked as not found by the AAIM.
- Mislabeled existing: The artery was present in the manual reference but at least a part of the labeled volume was inaccurate. A subjective decision in each case was used to determine whether the degree of mislabeling was clinically relevant.
- Mislabeled nonexisting: The artery was not present in the manual reference but was marked as existing by the AAIM.
- Not identified: The artery was not labeled, even though it did exist in the manual reference.
- Too short: The identified segment belonged to the correct artery but did not include the required segment.

#### Clinical Validation

For the UBA167 to be clinically relevant, it has to be applicable to vascular diseases. As a proof-of-concept of this generalizability, the UBA167 was applied to a clinical sample of ten patients with transient ischemic attacks or lacunar infarcts. The labeling was performed with the AAIM. Again, the labeled images were evaluated using the previously mentioned criteria. In addition, results were compared to a reference obtained from computed tomography (CT) angiograms.

## Results

**Fig. 3:**
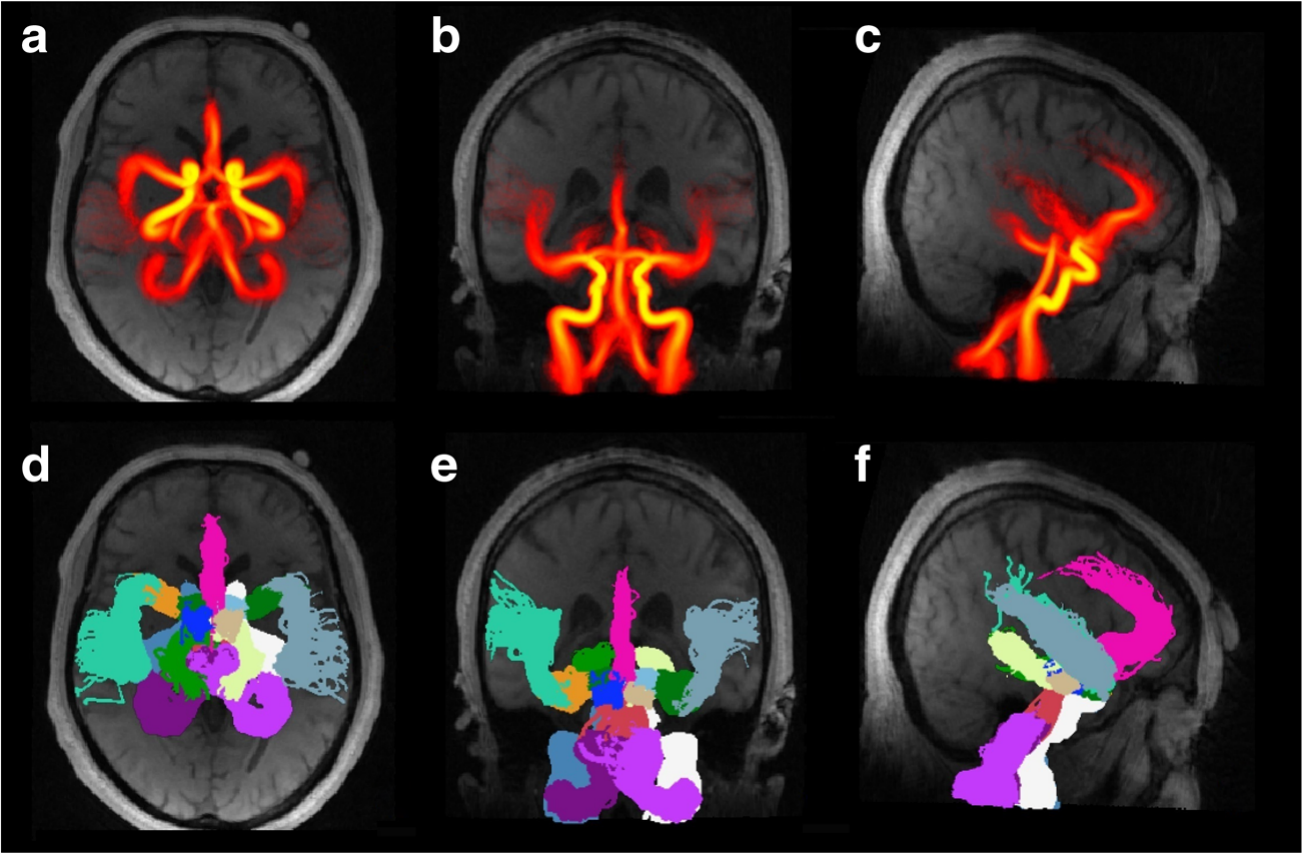
Visualization of the probabilistic (**a**–**c**) and artery - specific (**d**–**f**) properties of the UBA167 shown in axial, coronal and sagittal view. The probability values (**a**–**c**) are visualized with a heat map, min = 0, mid = 0.1 and max = 1.0. In (**d**–**f**), each voxel is labeled as the artery with the highest probability

**Fig. 4:**
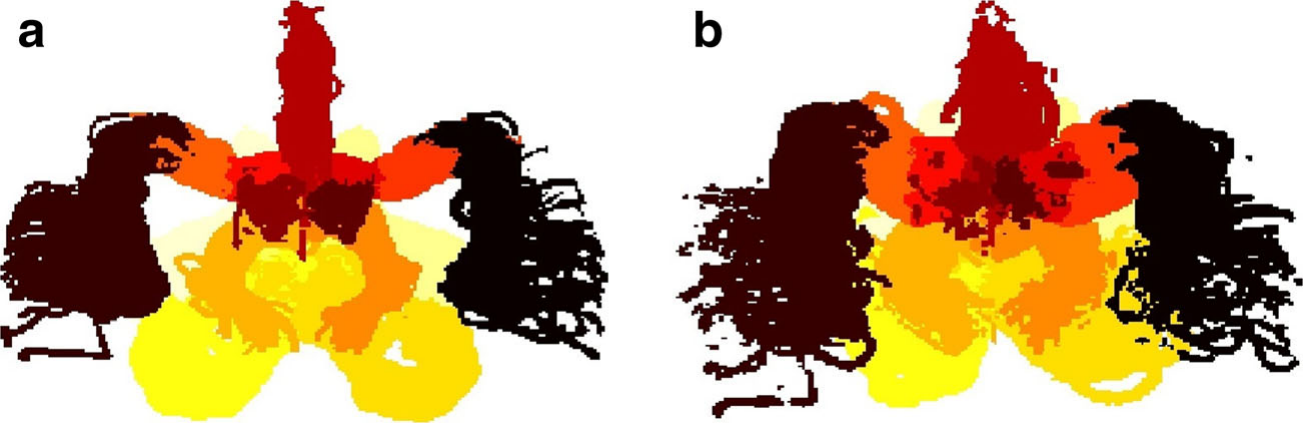
Visual comparison of the two atlases and the volumes of the probability maps. A maximum value projection of **a**) UBA167 and **b**) the rigid-body atlas. Each probability map is presented in a separate color

The arterial atlas, UBA167, is presented in Fig. 3, and the specific contribution of applying non-linear image normalization over rigid-body co-registration is seen in Fig. 4.

As revealed by the more confined probability maps, the non-linear image normalization generated a higher degree of arterial alignment than the rigid-body transformation.

### Prevalence of Arteries

The number of arteries included in UBA167 and their average volumes are presented in Table 1. In total 2360 arteries were manually labeled. ICA, MCA and ACA_distal_ were present in all subjects. BA and PCA were present in over 98 % of the subjects. VA, ACA and MCA_distal_ were present in at least 90 % of subjects, while only 38 % (63/167) had a PCoA on any side, and 28.7 % (48/167) had a fetal PCoA (missing P1).

**Table 1:** For each probability map, the number of included arteries and their average volumes are presented, as well as the concatenated volume, the ratio between concatenated volume and average arterial volume, the percentage of the concatenated volume where no other probability map had a higher value, and the maximum value of each probability map.

Artery	Number of arteries (Percent of subjects)	Average arterial volume ± SD [cm^3^]	Concatenated volume [cm^3^]		Arterial volume ratio (AVR)		Dominating volume [%]		Maximum probability value	
			UBA	RB	UBA	RB	UBA	RB	UBA	RB
Right ICA	167 (100)	3.0 ± 0.43	19.5	51.0	6.4	16.8	97.6	95.5	1.0	0.47
Left ICA	167 (100)	3.0 ± 0.52	19.8	45.8	6.7	15.5	97.1	95.2	1.0	0.53
BA	166 (99.4)	0.52 ± 0.18	9.9	18.7	19.0	35.7	72.5	73.1	0.79	0.25
Right VA	153 (91.6)	0.70 ± 0.43	18.3	36.4	26.1	52.0	95.4	95.2	0.51	0.12
Left VA	154 (92.2)	0.83 ± 0.41	19.2	39.7	23.2	48.0	91.8	93.5	0.68	0.15
Right PCA	166 (99.4)	0.29 ± 0.13	6.4	14.2	22.0	48.1	88.4	79.6	0.54	0.14
Left PCA	165 (98.8)	0.28 ± 0.14	6.2	13.0	21.9	46.1	89.6	82.7	0.58	0.15
Right MCA	167 (100)	0.46 ± 0.12	5.8	15.9	12.6	34.8	81.9	79.0	0.90	0.20
Left MCA	167 (100)	0.44 ± 0.13	6.5	16.3	14.8	37.1	81.6	78.2	0.89	0.18
Right ACA	157 (94.0)	0.24 ± 0.074	2.9	9.4	11.8	39.0	75.0	59.8	0.85	0.16
Left ACA	162 (97.0)	0.25 ± 0.078	2.5	9.2	10.0	36.4	64.4	50.9	0.83	0.18
ACA_distal_	167 (100)	0.59 ± 0.27	10.7	21.5	18.2	36.6	95.9	93.8	0.66	0.22
Right PCoA	50 (29.9)	0.17 ± 0.060	2.4	4.5	13.9	26.1	69.0	38.8	0.88	0.24
Left PCoA	30 (17.9)	0.19 ± 0.065	1.7	3.5	9.1	18.8	77.4	42.8	0.90	0.23
Right MCA_distal_	162 (97.0)	0.40 ± 0.25	14.8	24.4	36.8	61.0	97.7	96.9	0.31	0.15
Left MCA_distal_	160 (95.8)	0.29 ± 0.20	12.5	18.3	42.8	62.8	97.9	97.4	0.29	0.11

*UBA* UBA167, *RB* rigid - body atlas, *ICA* Internal carotid artery, *VA* Vertebral artery, *BA* Basilar artery, *PCA* Posterior cerebral artery, *MCA* Middle cerebral artery, *ACA* Anterior cerebral artery, *PCoA* Posterior communicating artery

### Arterial Volume Ratio

The AVR of each probability map is presented in Table 1 and reflects how well the normalization method works and to which extent each artery permits such normalization. A perfect normalization would result in an AVR of 1.0, and higher values indicate a less effective normalization. The AVR for the probability maps in UBA167 were significantly lower than for the rigid-body atlas (*p* < 0.001, Wilcoxon signed-rank test). When looking at the whole atlas, the AVR for the UBA167 was 13.7, and the corresponding value for the rigid-body atlas was 29.3.

In general, the proximal arteries of the anterior circulation (ICA, MCA, ACA and PCoA) had a low AVR (6.4 to 14.8) compared with posterior (PCA, VA and BA, AVR 19.0 to 26.1) and distal arteries (MCA_distal_ and ACA_distal_, AVR 18.2 to 42.8). The AVR was negatively correlated with the accuracy of the leave-one-out validation (*p* = 0.035, Spearman correlation, rho = − 0.46, one-tailed probability).

### Dominating Volume and Maximum Probability

The dominating volume of each probability map and the maximum probability value are also presented in Table 1. The average dominating volume for UBA167 was 85.8 %, compared with 74.9 % in the rigid-body atlas, and this difference was statistically significant (*p* < 0.005, Wilcoxon signed-rank test), indicating that the DARTEL normalization did indeed improve the spatial alignment between subjects.

The maximum probability value is also a measurement of spatial distribution. For UBA167, many arteries had voxels where over 80 % of subjects overlapped, ICA even had 100 % overlap in some voxels. For the rigid-body atlas, no more than 53 % of the subjects overlapped in any single voxel.

### Correctly Identifying Cerebral Arteries

The result from the leave-one-out validation can be found in Table 2. The average labeling accuracy for the leave-one-out validation was 96 %. Lower values were observed for VA, PCoA and left MCA_distal_. The specificity could not be calculated for all arteries because the number of true negatives was zero for most arteries.

**Table 2:** Labeling results from leave-one-out validation

Artery	Correctly identified existing (TP)	Correctly identified non-existing (TN)	Mislabeled non-existing (FP)	Mislabeled existing (FN)	Not identified (FN)	Too short (FN)	Sensitivity [%]	Specificity [%]	Accuracy [%]
Right ICA	165	0	0	2	0	0	99	–	99
Left ICA	167	0	0	0	0	0	100	–	100
BA	163	0	1	2	1	0	98	0	98
Right VA	136	4	11	7	1	9	89	27	84
Left VA	145	8	5	6	0	4	95	57	92
Right PCA	165	1	0	0	0	1	99	100	99
Left PCA	163	2	0	0	0	2	99	100	99
Right MCA	167	0	0	0	0	0	100	–	100
Left MCA	167	0	0	0	0	0	100	–	100
Right ACA	154	9	1	0	3	0	98	9	98
Left ACA	160	5	0	0	2	0	99	100	99
ACA_distal_	167	0	0	0	0	0	100	–	100
Right PCoA	43	112	5	0	7	0	86	96	93
Left PCoA	26	124	13	0	4	0	87	91	90
Right MCA_distal_	160	2	3	0	0	2	99	40	97
Left MCA_distal_	142	3	4	0	5	13	89	43	86

True positive (TP), false positive (FP), true negative (TN), and false negative (FN) rates, as well as sensitivity and specificity for identifying each artery with AAIM are also presented. Without any TN or FP, it is not possible to calculate specificity

*ICA* Internal carotid artery, *VA* Vertebral artery, *BA* Basilar artery, *PCA* Posterior cerebral artery, *MCA* Middle cerebral artery, *ACA* Anterior cerebral artery, *PCoA* Posterior communicating artery

The average labeling accuracy in the clinical sample was 92.5 % (Table 3). In two patients, the identified right PCA was too short to fulfill the evaluation criteria. This was also the case for the right VA in two patients. One ACA_distal_ and one MCA_distal_ on each side were not identified. The existing PCoAs were not identified in the clinical sample. In the CT-angiography that serves as the reference in this evaluation, PCoA was found bilaterally in one subject and unilaterally in three subjects.

**Table 3:** Labeling results for the clinical validation

Artery	Correctly identified existing (TP)	Correctly identified non-existing (TN)	Not identified (FN)	Too short (FN)	Sensitivity [%]	Specificity [%]	Accuracy [%]
Right ICA	10	0	0	0	100	–	100
Left ICA	10	0	0	0	100	–	100
BA	10	0	0	0	100	–	100
Right VA	8	0	0	2	80	–	80
Left VA	10	0	0	0	100	–	100
Right PCA	8	0	0	2	80	–	80
Left PCA	10	0	0	0	100	–	100
Right MCA	10	0	0	0	100	–	100
Left MCA	10	0	0	0	100	–	100
Right ACA	10	0	0	0	100	–	100
Left ACA	10	0	0	0	100	–	100
ACA_distal_	9	0	1	0	90	–	90
Right PCoA	0	7	3	0	0	100	70
Left PCoA	0	8	2	0	0	100	80
Right MCA_distal_	9	0	1	0	90	–	90
Left MCA_distal_	9	0	1	0	90	–	90

True positive (TP), false positive (FP), true negative (TN), and false negative (FN) rates, as well as sensitivity and specificity for identifying each artery with AAIM are also presented. Without any TN or FP, it is not possible to calculate specificity

*ICA* Internal carotid artery, *VA* Vertebral artery, *BA* Basilar artery, *PCA* Posterior cerebral artery, *MCA* Middle cerebral artery, *ACA* Anterior cerebral artery, *PCoA* Posterior communicating artery

## Discussion

The probabilistic atlas Umeå Brain Arteries (UBA167), which contains 16 major brain arteries, was constructed based on manually labeled 4D flow MRI angiography from 167 subjects. This is the first project describing the “artery-specific” 3D spatial distribution within the human brain. UBA167 can be used to automatically label and segment the major cerebral arteries for future automated blood flow quantification. Similar to successful brain tissue atlases (Diedrichsen et al. 2009; Tzourio-Mazoyer et al. 2002; van Baarsen et al. 2016), UBA167 is provided in MNI space. We believe that multiple additional neuroimaging applications can be developed around this atlas. For example, the atlas can be used to extract geometrical measures such as size and tortuosity of brain arteries. In addition, the atlas can be used to detect and correct for signals in large arteries that can degrade the physiological interpretation of MRI data (e.g. arterial spin labeling, functional MRI, diffusion imaging). For example, in functional MRI, removal of vascular contamination is of known importance (Kiviniemi et al. 2003).

Achieving sufficient spatial inter-individual alignment of brain arteries cannot be taken for granted, as demonstrated by the fact that DARTEL normalization outperformed rigid-body transformation. This finding is analogous to results obtained for brain structural normalization (Klein et al. 2009). In the current paper we hypothesized that normalization prior to atlas labeling would provide a feasible pipeline for automated labeling. Our results supported this hypothesis by showing that all major arteries were labeled with high accuracy (>95 %), indicating that the achieved inter-individual spatial variability permitted efficient atlas construction.

UBA167 was constructed from a population-based sample (Nevalainen et al. 2015), and can thus be expected to represent a wide range of vascular morphology. This may have contributed to our finding of a high accuracy when using UBA167 on the ten stroke patients. An atlas based on a more selected sample (for instance only using subjects without hypertension, hyperlipidemia and obesity) could have resulted in less generalizability as such cardiovascular risk factors may affect the anatomy and tortuosity of cerebral vessels (Bullitt et al. 2009; Hiroki et al. 2002).

The atlas is developed from data on individuals from a limited age span. We still expect a high degree of generalizability of the atlas, assuming that future studies normalize data according to the present study. However, future studies using the atlas have to verify its functionality for that particular sample.

The main innovation of the UBA167 is the combination of the artery-specific probabilistic properties with the fully automatic labeling of the major cerebral arteries. The UBA167 is thus prepared for automatic blood flow quantification in 4D flow MRI data. Pioneering work on characterization of the cerebral arterial system has been based on small sample sizes (Nowinski et al. 2009a, 2011, 2013) or has not included specific probabilities for individual arterial segments (Forkert et al. 2013; Mut et al. 2014; Wright et al. 2013). Knowledge of vascular morphology has also been used for semi-automated segmentation and labeling (Bogunović et al. 2012; Ghanavati et al. 2014; Passat et al. 2006).

Our finding of a maximum probability value of 1.0 in the bilateral ICA means that there exist one or more voxels in each ICA where all 167 subjects overlapped. These voxels provide the compelling option to explore seeding points, combined with region-growing segmentation schemes, since the probability that those voxels will be within the ICA for a new subject is very high (Passat et al. 2005). UBA167 as a whole can also be used to specify the region of interest or provide spatial information for arterial segmentation to reduce computational time (Passat et al. 2006).

UBA167 enabled a high labeling accuracy (Tables 2 and 3). Although the input data for the labeling validation was from 4D flow MRI, a comparable imaging technique can be expected to produce equally accurate results when combined with the AAIM.

We used a velocity encoding of 110 cm/s for the 4D flow sequence. This value was selected to avoid aliasing in large arteries. This option is not optimal to visualize and quantify the slow-velocity blood flow of the posterior communicating cerebral arteries (Dunås et al. 2016). Indeed, this was clearly evident when the CT angiogram images from the patient group were reviewed. Here CT angiography detected additional PCoA arteries that were not visible on the thresholded AI. This is not a strict limitation of UBA167 and labeling procedure per se, but rather a manifestation of differences in the underlying measurement techniques. However, with respect to the development of an automatic flow assessment of 4D flow MRI data, the high labeling accuracy for arteries that had detectable velocities was very promising. Future developments could improve the labeling accuracy of VA segments by improving the labeling criteria. The evaluation conditions for the labeling were set to ensure that segments needed for future blood flow quantification were correctly identified, therefore some existing arteries could be marked as too short.

The UBA167 included the major cerebral arteries, therefore it does not allow investigation of more distal arteries. Such distal arteries could also be added, but the increasing inter-individual variations in anatomy at that depth in the arterial tree would potentially limit the usefulness of such expansions. This effect can be seen in the MCA_distal_ in Fig. 3, appearing with lower spatial alignment between subjects compared with more proximal arteries. Since the P1 and P2 segments were defined as the same artery, and the anterior communicating artery was not included, UBA167 cannot differentiate between some of the typical morphological variants. The standard variations that can be automatically identified are missing A1 or PCoA (Krabbe-Hartkamp et al. 1998).

## Conclusion

UBA167 is an artery-specific probabilistic atlas based on 16 manually-labeled major cerebral arteries from 167 subjects. The UBA167 enables a high accuracy in automatic arterial labeling in both population-based subjects and in ischemic patients. Comparison to rigid-body alignment showed a large improvement in spatial alignment for non-linear normalization. Taken together, this study provides compelling first evidence for the usefulness of a probabilistic stereotactic atlas of the major cerebral arteries.

## Information Sharing Statement

The atlas (RRID:SCR_016319) will be made publically available at time of publication at http://www.nitrc.org/projects/brainarteries

## Abbreviations

AAIM: Automatic atlas based Artery Identification Method
ICA: Internal carotid artery
VA: Vertebral artery
BA: Basilar artery
PCA: Posterior cerebral artery
MCA: Middle cerebral artery
ACA: Anterior cerebral artery
PCoA: Posterior communicating artery
AI: Angiographic image
AVR: Arterial volume ratio
MNI: Montreal Neurological Institute
